# Gene Replacement in *Mycobacterium chelonae*: Application to the Construction of Porin Knock-Out Mutants

**DOI:** 10.1371/journal.pone.0094951

**Published:** 2014-04-16

**Authors:** Vinicius Calado Nogueira de Moura, Sara Gibbs, Mary Jackson

**Affiliations:** Mycobacteria Research Laboratories, Department of Microbiology, Immunology and Pathology, Colorado State University, Fort Collins, Colorado, United States of America; Centre National de la Recherche Scientifique - Université de Toulouse, France

## Abstract

*Mycobacterium chelonae* is a rapidly growing mycobacterial opportunistic pathogen closely related to *Mycobacterium abscessus* that causes cornea, skin and soft tissue infections in humans. Although *M. chelonae* and the emerging mycobacterial pathogen *M. abscessus* have long been considered to belong to the same species, these two microorganisms considerably differ in terms of optimum growth temperature, drug susceptibility, pathogenicity and the types of infection they cause. The whole genome sequencing of clinical isolates of *M. chelonae* and *M. abscessus* is opening the way to comparative studies aimed at understanding the biology of these pathogens and elucidating the molecular bases of their pathogenicity and biocide resistance. Key to the validation of the numerous hypotheses that this approach will raise, however, is the availability of genetic tools allowing for the expression and targeted mutagenesis of genes in these species. While homologous recombination systems have recently been described for *M. abscessus*, genetic tools are lacking for *M. chelonae*. We here show that two different allelic replacement methods, one based on mycobacteriophage-encoded recombinases and the other on a temperature-sensitive plasmid harboring the counterselectable marker *sacB*, can be used to efficiently disrupt genes in this species. Knock-out mutants for each of the three porin genes of *M. chelonae* ATCC 35752 were constructed using both methodologies, one of which displays a significantly reduced glucose uptake rate consistent with decreased porin expression.

## Introduction

Rapidly growing non-tuberculous mycobacteria (RGM) are opportunistic pathogens widespread in the environment that can cause a wide spectrum of infections [1]. Among RGM, the *Mycobacterium chelonae-abscessus* group is considered to be the most pathogenic for humans [1], [2]. On the basis of their almost identical biochemical features, *M. chelonae* and *M. abscessus* were originally considered to belong to the same species (‘*M. chelonei*’ or ‘*M. chelonae*’). Advances in molecular biology techniques then led to their separation into two distinct species in 1992 [1], [3]. *M. abscessus* has since been shown to consist of three subspecies, *M. abscessus* subsp. *abscessus*, *M. abscessus* subsp. *massiliense* and *M. abscessus* subsp. *bolletii* [4], [5], even though *massiliense* and *bolletii* have recently been proposed to represent one single subspecies [6]. Distinguishing between *M. chelonae* and *M. abscessus* (*sensu lato*) is important because these organisms are typically not associated with the same types of infections in humans, differ in their pathogenicity and show very different antimicrobial susceptibility patterns [2], [7]. *M. abscessus* (*sensu lato*) is the most pathogenic and antibiotic-resistant RGM. It is responsible for more than 80% of all pulmonary infections caused by RGM in the United States [1], [8] and it is also associated with contaminated traumatic skin wounds, post-surgical soft tissue infections, central nervous system infections and disseminated infections in immunodeficient patients [9]–[11]. *M. chelonae* in contrast is only rarely a cause of chronic lung disease [8] and is better known as a causative agent of cornea, skin and soft tissue infections [1]. Pulmonary infections with *M. abscessus* (*sensu lato*) have been increasing in prevalence throughout the world, particularly in patients with structural lung disease such as chronic obstructive pulmonary disease, bronchiectasis, and cystic fibrosis [2], [12], [13]. Patient-to-patient transmission involving *M. abscessus* subsp. *massiliense* has recently been proposed [13], [14]. Another important distinction between *M. chelonae* and *M. abscessus* is their optimum growth temperature. The temperature optimum of *M. chelonae* is 28–30°C whereas *M. abscessus* (*sensu lato*) grows best at 35–37°C.

The first genome sequence of *M. abscessus* subsp *abscessus* (strain ATCC 19977) was completed and released in 2009 [15], followed by that of several other *M. abscessus* subsp. *abscessus*, *massiliense* and *bolletii* isolates. The genome sequencing of the two first *M. chelonae* isolates (strains ATCC 35752 and 1518) is in progress. Comparative genomics of these two closely related RGM is expected to provide significant insights into the molecular bases underlying the important differences associated with the physiology, pathogenicity and drug susceptibility of RGM. A prerequisite to the validation of the numerous hypotheses that comparative genomics will raise, however, is the availability of expression and mutagenesis systems allowing for the knock-in and knock-out of genes in these microorganisms.

Progress was made recently toward the development of conditional expression and homologous recombination systems for *M. abscessus*. Although orders of magnitude less efficient than in *M. smegmatis*, these methodologies were successfully used to construct a null and a conditional mutant [16], [17]. Foremost among the obstacles that genetic strategies have to overcome in RGM pathogens in general is their low electrotransformation efficiency, intrinsic high level of resistance to antibiotics and propensity to develop spontaneous resistance to commonly used antibiotic resistance markers, and relatively narrow temperature growth range limiting the effectiveness of counterselectable temperature-sensitive plasmids and phages [16], [17]. To the best of our knowledge, no mutagenesis system has yet been optimized for *M. chelonae*. With the goal of facilitating future studies on the biology of RGM pathogens, we here undertook to address the current lack of suitable genetic systems in *M. chelonae*. Various selectable and counterselectable markers and allelic replacement methods, namely the temperature sensitive – SacB (*Ts-SacB*) system [18], the thermosensitive mycobacteriophage system [19] and the recombinase-based system [20] were tested and compared. Both the Ts-sacB and the recombinase-based systems were found to be functional in *M. chelonae* as long as appropriate selectable markers are used.

## Materials and Methods

### Bacterial strains and culture media

*Escherichia coli* DH5α, the strain used for cloning, was grown in LB Lennox (BD, Difco) medium at 37°C. *M. chelonae* strains ATCC 35752 and 9917 [21], and *M. abscessus* ATCC 19977 were grown in LB or Middlebrook 7H9-OADC broth (BD, Difco) supplemented with 0.05% Tween 80, 7H11-OADC agar (BD, Difco) or minimal Sauton's medium supplemented with 0.05% tyloxapol. Kanamycin (Kan) and Gentamicin (Gen) were added to final concentrations of 200 µg/ml. Zeocin (Zeo) was added to a final concentration of 100 µg/ml.

### Electrotransformation

Electrocompetent *M. chelonae* and *M. abscessus* cells were prepared by washing bacterial pellets two times with double distilled water containing 0.05% Tween 80 and one time with 10% glycerol–0.05% Tween 80 at 4°C. One hundred microliter aliquots of freshly prepared competent cells in 10% glycerol–0.05% Tween 80 were electroporated in the presence of 1 µg of plasmid DNA or 300 ng of linear DNA in 0.2-cm cuvettes with a single pulse (2.5 kV; 25 µF; 1,000 ohms). One milliliter of fresh 7H9-OADC-Tween 80 medium was then added, and the culture was incubated at 30°C (*M. chelonae*) or 37°C (*M. abscessus*) for 4 h before plating on 7H11-OADC agar plates.

### Allelic replacement using the *Ts-SacB* system

The *MCH_4689c* gene and flanking regions was PCR-amplified from *M. chelonae* ATCC 35752 genomic DNA with primers MCH4689Fw2 (5′-TTTTTCTAGAGTGTATCGGCTGCGAGTTAGC-3′) and MCH4689Rv2 (5′-TTTTTCTAGAGCGGGTGGTAATGGTCGCAT-3′) and the disrupted alleles, *MCH_4689c::kan* and *MCH_4689c::zeo*, were obtained by inserting either the *Tn903* kanamycin resistance cassette from pUC4K (GE Healthcare) or the zeocin resistance cassette from pEM7/Zeo (Invitrogen) at the unique SgfI restriction site of *MCH_4689c*. Following similar strategies, the *MCH_4690c* and *MCH_4691c* genes and their flanking regions were PCR-amplified individually from *M. chelonae* ATCC 35752 genomic DNA using the following primer combinations: MCH4690Fw (5′-TTTTTCTAGATTACGTAGGTCGAGGCGCCG-3′) and MCH4690Rv (5′-TTTTTCTAGACGTCCCGATCACTGCCAGCC-3′) (for *MCH_4690c*); and MCH4691Fw (5′-TTTTTCTAGAGTCTTGGGTTCGGCTAACTTC-3′) and MCH4691Rv (5′-TTTTTCTAGATGTACGAAGCCTCAGGACCA-3′) (for *MCH_4691c*). Disrupted alleles of *MCH_4690c* and *MCH_4691c* were obtained by insertion of the *kan* or *zeo* resistance cassettes at the unique SgfI site of these genes. All six disrupted alleles were then cloned in the XbaI-cut pPR27-xylE [18], [22], yielding pPR27-4689-KX, pPR27-4689-ZX, pPR27-4690-KX, pPR27-4690-ZX, pPR27-4691-KX and pPR27-4691-ZX, the plasmids used to achieve allelic replacement at the *MCH_4689c*, *MCH_4690c* and *MCH_4691c* loci of *M. chelonae* (Table 1).

**Table 1:** Strains and plasmids used in this study.

Plasmid or strain	Features	Source or reference
Strains		
*M. chelonae* ATCC 35752	*M. chelonae* reference strain	ATCC
*M. chelonae* 9917	Glutaraldehyde-resistant isolate of *M. chelonae*	[21]
*M. abscessus* ATCC 19977	*M. abscessus* reference strain	ATCC
Plasmids		
pOMK	pBluescript KS- derivative carrying a mycobacterial origin of replication and a Kan resistance gene	[43]
pOMK-zeo	pOMK derivative carrying the *zeo* resistance gene from pEM7/Zeo	This study
pOMK_4691	pOMK derivative expressing *MCH_4691c* under control of its own promoter	This study
pOMK_[4691_4689]	pOMK derivative expressing *MCH_4691c*, *MCH_4690c* and *MCH_4689c* under control of their own promoter	This study
pJV53	Recombineering plasmid carrying the Che9c *gp60* and *gp61* genes under control of the acetamidase promoter	[20]
pJV53-xylE	pJV53 derivative carrying the *xylE* gene	This study
pEM7/Zeo	Plasmid carrying the Zeo resistance cassette	Invitrogen
pUC4K	Plasmid carrying the Kan resistance cassette	GE Healthcare
pPR27-xylE	Mycobacterial shuttle plasmid carrying the *sacB* counterselectable marker, a Gen resistance cassette and *xylE*	[22]
pPR27-4689-KX	pPR27-xylE derivative carrying *MCH4689c::kan*	This study
pPR27-4690-KX	pPR27-xylE derivative carrying *MCH4690c::kan*	This study
pPR27-4691-KX	pPR27-xylE derivative carrying *MCH4691c::kan*	This study
pPR27-4689-ZX	pPR27-xylE derivative carrying *MCH4689c::zeo*	This study
pPR27-4690-ZX	pPR27-xylE derivative carrying *MCH4690c::zeo*	This study
pPR27-4691-ZX	pPR27-xylE derivative carrying *MCH4691c::zeo*	This study

The pPR27-xylE constructs carrying the disrupted porin genes were electroporated in *M. chelonae* ATCC 35752 and transformants were selected on Kan or Zeo-containing medium at 30°C. One to three XylE positive colonies (i.e., turning yellow upon exposure to catechol) [22] were inoculated in 7H9-OADC-Tween 80 broth at 30°C for 5–7 days and finally plated onto 7H11-OADC containing Kan or Zeo and 10% sucrose at 37°C. Colony forming units (CFU) displaying the expected phenotype for allelic exchange mutants (i.e., resistance to sucrose and Kan or Zeo, XylE^−^) were picked after 14 to 21 days and analyzed by PCR using primers MCHDCO1 (5′-ATCTGGCAGGTCGCGAAGTC-3′) and MCHDCO2 (5′-ACCGGAGATATCGTCGACATC-3′) to amplify the entire porin gene cluster (Fig. S1). That allelic replacement occurred at the right porin locus was confirmed by sequencing the regions immediately flanking the antibiotic resistance cassette.

pOMK_4691 and pOMK_[4691_4689], the constructs used for complementation of the *MCH_4691c* knock-out mutant, were constructed by cloning the *MCH_4691c* gene or the entire porin gene cluster encompassing *MCH_4691c, MCH_4690c* and *MCH_4689c* plus 169 bp of upstream and 522 bp (pOMK_[4691_4689]) to 525 bp (pOMK_4691) of downstream DNA sequence into the XbaI restriction site of pOMK (Table 1).

### Allelic replacement using the recombineering system

This system consists of performing allelic exchange in strains expressing the gp60 and gp61 recombineering proteins from mycobacteriophage Che9c that effectively promote homologous recombination in mycobacteria [20]. The *gp60* and *gp61*genes are expressed from the replicative plasmid pJV53 under control of an acetamide-inducible promoter [20]. Acetamide-induced strains harboring pJV53 are electro-transformed with allelic exchange substrates under the form of linear DNA and double crossover mutants are isolated on selective medium.

To facilitate the detection of pJV53 transformants, a derivative of pJV53 carrying the colored marker *xylE* was first constructed by cloning the *xylE* gene from pPR27-xylE in the unique SpeI restriction site of pJV53 (Table 1). pJV53-xylE was introduced in *M. chelonae* ATCC 35752 by electroporation and a kanamycin-resistant-XylE^+^ transformant was then cultured at 30°C in LB broth containing Kan, 0.05% tyloxapol and 0.2% succinate to mid-log phase (A_600nm_ = 0.4) at which point 0.2% acetamide was added. After 3 h of induction, electrocompetent cells were prepared as described above. The competent cells were transformed with 300 ng of linear allelic exchange substrates. The linear *zeo* cassette-disrupted alleles used to achieve gene replacement at the *MCH_4689c*, *MCH_4690c* and *MCH_4691c* loci of *M. chelonae* ATCC 35752 were identical to those used in the *Ts-sacB* system. Candidate allelic exchange mutants were analyzed by PCR and sequencing as described above (Fig. S1).

### Drug susceptibility testing

MIC values were determined in 7H9-OADC-Tween 80 and Muller-Hinton II broth (BD, Difco) in a total volume of 200 µl in 96-well microtiter plates. *M. chelonae* cultures grown to early log phase (A_600nm_ = 0.2) were diluted to a final concentration of 5×10^e5^ CFU/ml and incubated in the presence of serial dilutions of the drugs for 5 to 7 days at 30°C. MICs were determined using the colorimetric resazurin assay and confirmed by visually scanning for growth.

### Glucose uptake experiment

[U-^14^C]glucose uptake experiments were essentially carried out as described by Stahl *et al*. [23]. Briefly, 100 ml *M. chelonae* cultures grown to an OD_600_ of 0.5 were harvested by centrifugation, washed twice in uptake buffer (50 mM Tris-HCl pH 7.1, 15 mM KCl, 10 mM NH_4_SO_4_, 1 mM MgSO_4_, 0.1% Tween 80), and resuspended in 25 ml of the same buffer. [U-^14^C]glucose (specific activity, 5 mCi/mmol, American Radiolabeled Chemicals) was mixed with cold glucose and added to the cell suspension to a final concentration of 20 µM. The mixtures were incubated at 30°C and 200 µl samples were removed at times ranging from 1 to 128 min. The cells were then added 200 µl of killing buffer (10% formalin: 0.1 M LiCl; 2∶1 by vol.) before being filtered through a 0.45 µm pore size membrane filter, washed twice with killing buffer and counted in a liquid scintillation counter. The uptake of glucose was expressed as pmol/mg (dry weight) cells. Glucose uptake experiments were performed twice using independent culture batches.

## Results and Discussion

### Porin genes as candidates for allelic replacement experiments

The particular structure and composition of the mycobacterial cell envelope is generally thought to be one of the major determinant of the intrinsic resistance of mycobacteria to most common antibiotics and other biocides [24], [25]. The presence of a highly impermeable outer membrane [26], [27] implies the existence of pore proteins spanning the outer membrane for the uptake of nutrients and efflux of waste products [28]. The Msp porins of the rapidly growing *Mycobacterium* species, *M. smegmatis*, represent the main general diffusion pathway for small hydrophilic molecules across the outer membrane of this bacterium [28], [29]. Msp porins have been extensively studied and showed to not only be major determinants of the susceptibility of *M. smegmatis* to multiple antibiotics and biocides [28], [30]–[33], but to also impact its vulnerability to killing by reactive nitrogen intermediates inside host phagocytic cells [34], [35]. As in other bacteria [36], porin expression in *M. smegmatis* appears to be tightly regulated [23], [37]. Whereas Msp-type porins are apparently not found in slow growing mycobacteria, the availability of a growing number of genome sequences from various *Mycobacterium* species and earlier biochemical and DNA hybridization studies indicate that they are likely to be widespread among RGM [28], [38]. Incidentally, the first Gram positive porin ever reported was that of an *M. chelonae* isolate [39], [40]. Evidence to date therefore points to an important role of porins in the physiology, biocide resistance and intracellular survival of RGM and the availability of isogenic knock-out mutants of *M. chelonae* deficient in their expression will prove useful for subsequent studies on the biology of this opportunistic pathogen.

**Figure 1:**
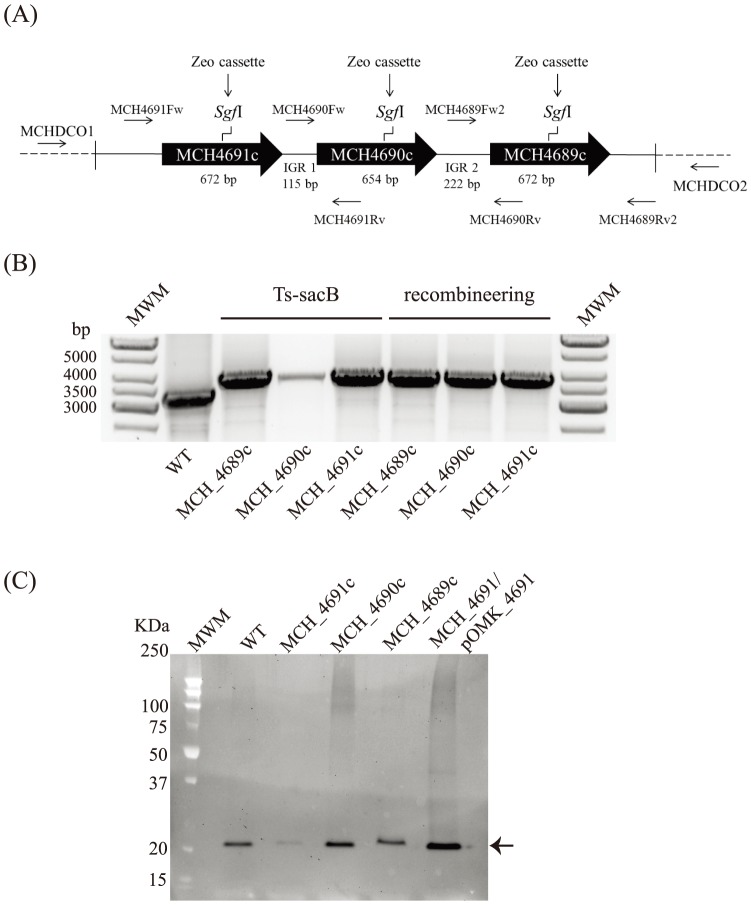
Gene replacement at the *MCH_4689c*, *MCH_4690c* and *MCH_4691c* porin loci of *M. chelonae* ATCC 35752 using the *Ts-sacB* and recombineering systems. (A) Porin gene cluster of *M. chelonae* ATCC 35752. The positions of the primers used to generate the allelic exchange substrates and analyze the candidate mutants are indicated. IGR1 and IGR2 represent the intergenic regions. (B) Candidate mutants obtained for each of the porin genes using the *Ts-sacB* or the recombineering systems were analyzed by PCR as described under Materials and Methods and confirmed by sequencing the regions flanking the resistance cassette. The expected size of the PCR fragments is 3.3 kb for the wild-type parent strain and 3.8 kb for the knock-out mutants. MWM, molecular weight marker. WT, wild-type. (C) Immunoblot analysis of porin production in the wild-type, mutant and complemented mutant strains. Strains were grown in 7H9-OADC-Tween 80 broth at 30°C to mid-log phase (OD600 = 1) and porins were selectively extracted from whole cells at 100°C using 0.5% *n*-octylpolyoxyethylene as a detergent as described [44]. Protein samples prepared from the same amount of cells for each strain were denatured by boiling in 80% DMSO followed by acetone precipitation [23]. Denatured proteins were loaded volume to volume, separated by SDS-PAGE, blotted onto a nitrocellulose membrane, and porins were detected using rabbit antiserum to purified MspA [23]. Immune complexes were detected by chemiluminescence (Pierce, ELC) and semi-quantified using the Image Lab software (Biorad).

Our previous work identified three porin genes (*MCH_4689c*, *MCH_4690c* and *MCH_4691c*) clustered on the chromosomes of the *M. chelonae* strain ATCC 35752 (Fig. 1A) and a glutaraldehyde-resistant *M. chelonae* isolate, 9917 [21]. 115- and 222-bp of intergenic space separate *MCH_4691c* from *MCH_4690c* and *MCH_4690c* from *MCH_4689c*, respectively, suggesting that these genes are expressed as single transcriptional units. MCH_4689c, MCH_4690c and MCH_4691c from *M. chelonae* ATCC 35752 differ from one another at one to four positions of the mature proteins (Fig. S2). Allelic exchange substrates were generated by inserting an antibiotic (kanamycin or zeocin) resistance cassette at the unique SgfI restriction site of each of the three porin genes of *M. chelonae* ATCC 35752 as described under Materials and Methods.

### Choice of allelic exchange methodologies

Mainly three homologous recombination systems have been used thus far in mycobacteria: (i) The *Ts-sacB* system, based on the use of a temperature-sensitive plasmid carrying the counterselectable marker *sacB* [18]; (ii) the temperature-sensitive mycobacteriophage system [19]; and the recombineering system [20]. *Ts-SacB* allows for allelic exchange substrates to be delivered on a replicative plasmid harboring a mycobacterial temperature-sensitive origin of replication and therefore to persist inside the cells until grown at a non-permissive temperature. Growth at non-permissive temperature in the presence of sucrose selects for allelic exchange mutants [18]. The recombineering system takes advantage of the transient expression of highly active mycobacteriophage-encoded recombinases to promote homologous recombination between the chromosomal copy of a target gene and a disrupted copy of this gene delivered to the cells as a linear DNA substrate [20]. The mycobacteriophage system compensates for the low transformation efficiency of mycobacteria by allowing allelic exchange substrates to be delivered to the cells with high efficiency on conditionally replicating mycobacteriophages. Thus far, the only two conditionally (temperature-sensitive) replicating phage systems available for this purpose are based on the mycobacteriophages TM4 and D29 [19]. Earlier reports indicated that none of these two mycobacteriophages efficiently transfect *M. chelonae* isolates [19], [41]. Our own assays confirmed these findings in the reference *M. chelonae* strain ATCC 35752 in that no plaques were obtained upon transfection of this isolate with high titers of TM4 or D29. Therefore, our efforts focused on the construction of porin knock-out mutants of *M. chelonae* using the recombineering and *Ts-sacB* systems.

### Transformation efficiency and spontaneous resistance to antibiotics

Key to the success of any allelic replacement methodology is the availability of selection markers allowing for the efficient selection of transformants and recombinant strains resulting from single or double crossover events. Given the high frequencies of spontaneous antibiotic-resistance mutations reported in *M. abscessus* subsp. *abscessus* [16], we first set out to test and compare different antibiotic selection markers (*gen*, *zeo* and *kan*) in *M. chelonae*. *M. chelonae* ATCC 35752 being intrinsically highly resistant to hygromycin (MIC>500 µg/ml), ampicillin (MIC>500 µg/ml), and streptomycin (MIC>200 µg/ml), the corresponding resistance cassettes were not tested here. *M. chelonae* ATCC 35752 was transformed with plasmids pOMK-zeo, conferring resistance both to Kan and Zeo, and pJV53-xylE conferring resistance to Kan (Table 1). In addition, the pPR27-based plasmids harboring *zeo* or *kan*-disrupted alleles of the porin genes were used. For comparison purposes, some plasmids were also transformed in *M. chelonae* 9917, although, in this case, the high intrinsic level of resistance of this particular isolate to zeocin (MIC>200 µg/ml compared to an MIC of 50 µg/ml for ATCC 35752) precluded selection on Zeo-containing media.

Transformation efficiencies in *M. abscessus* ATCC 19977 were in line with those reported previously [16]. Transformation efficiencies in *M. chelonae* ATCC 35752 were two orders of magnitude greater than that measured for *M. abscessus* ATCC 19977 with pOMK-zeo, and considerably greater (>400-fold) with pOMK-zeo and pJV53-xylE than with the pPR27-derived constructs (Table 2). Compared to the ATCC 35752 strain, transformation efficiencies were 70 to a 500-fold less in the 9917 isolate, suggestive of important variations in the transformation efficiency of different *M. chelonae* isolates (Table 2). All *M. chelonae* transformants harboring *xylE*-containing plasmids (pJV53-xylE; pPR27-derived constructs) stained yellow upon spraying with catechol [22] indicating that XylE is a functional colored marker in this species. Interestingly, these transformations also revealed highly variable selection efficiencies for the *kan* cassette depending on the plasmid used (Table 2). Whereas Kan selection was very efficient with pOMK-zeo and pJV53-xylE in *M. abscessus* ATCC 19977, *M. chelonae* ATCC 35752 and *M. chelonae* 9917 (56 to a 100% of the tested Kan^R^ colonies contained the plasmids), less than 1% of the selected Kan^R^ *M. chelonae* ATCC 35752 CFUs actually contained plasmids when pPR27-based constructs were used. The selection efficacy of Gen upon transformation of pPR27-based constructs was 5 to 20-fold greater than that of Kan (Table 2). Overall, and similar to the situation in *M. abscessus* ATCC 19977 [16], selection with *zeo* was consistently the most efficient independent of the type of plasmid used (Table 2). That the variable selection efficacy of *kan* was due to the origin of replication and copy number of the plasmids is unlikely given that all of them harbor the mycobacterial origin of replication from pAL5000 (albeit, a temperature-sensitive version of it in the pPR27 plasmids). Most noticeable is the different sizes of the plasmids, which are greater than 12 kb for the pPR27 constructs compared to 7.7 kb for pOMK-zeo and 9.8 kb for pJV53-xylE. This difference in size may account, at least in part, for the lower transformation efficiencies observed with the larger pPR27 plasmids (Table 2).

**Table 2:** Comparative electrotransformation efficiency and spontaneous resistance to different antibiotics in *M. chelonae* strains ATCC 35752 and 9917, and *M. abscessus* ATCC 19977.

Transformant	*M. chelonae*	*M. chelonae*	*M. abscessus*
	ATCC 35752	9917	ATCC 19977
pOMK-zeo	Kan: 2.3×10^5^	Kan: 394	Kan: 3.0×10^3^
	[100%]	[100%]	[100%]
	Zeo: 2.8×10^5^		Zeo: 3.6×10^3^
	[100%]		[100%]
pPR27-4689-ZX	Zeo: 9 [100%]	nd	nd
	Gen: 32 [20.3%]		
pPR27-4690-ZX	Zeo: 4 [100%]	nd	nd
	Gen: 27 [27.1%]		
pPR27-4691-ZX	Zeo: 5 [100%]	nd	nd
	Gen: 3 [17.2%]		
pPR27-4689-KX	Kan: 335 [0.9%]	nd	nd
	Gen: 18 [16.6%]		
pPR27-4690-KX	Kan: 263 [0.8%]	nd	nd
	Gen: 18 [5.6%]		
pPR27-4691-KX	Kan: 311 [1.0%]	nd	nd
	Gen: 23 [13.0%]		
pJV53-xylE	Kan: 2.1×10^3^	Kan: 30	Kan: 2.5×10^3^
	[56.8%]	[100%]	[99.2%]

Transformation efficiencies upon selection with the indicated antibiotics on 7H11-OADC agar are expressed as numbers of drug-resistant CFUs per µg of DNA electroporated. The percentage below each transformation efficiency value represents the percentage of Kan, Zeo or Gen-resistant CFUs confirmed to be actual transformants either by PCR (pOMK-zeo) or determination of their XylE phenotype (all other plasmids).

Taken together, the results of the experiments presented in Table 2 indicated that: (i) transformation efficiencies of *M. chelonae* vary with the isolate and the plasmid transformed but are up to 100 times greater than that of *M. abscessus* ATCC 19977; (ii) Gen, Kan and Zeo can all be used as selection markers in *M. chelonae* ATCC 35752; however, Zeo seems to be the least prone to spontaneous resistance; (iii) XylE is a functional colored marker in *M. chelonae*.

### Construction of allelic exchange mutants using the *Ts-sacB* system

Both *kan* and *zeo*-disrupted alleles of the porin genes were constructed for use in allelic exchange experiments with the *Ts-sacB* system (Table 1). Attempts to grow *M. chelonae* ATCC 35752 at increasing temperatures set the maximum at which the temperature-sensitive pPR27 plasmids could be counterselected at about 37°C since no colonies formed on 7H11-OADC plates beyond this temperature (39°C). *M. chelonae* ATCC 35752 colonies appeared after 6 days at 37°C instead of 3 days at 30°C.

Transformation of *M. chelonae* ATCC 35752 with pPR27-4691-ZX followed by plating on 7H11-OADC-Zeo at 30°C or 37°C in the presence of absence of sucrose in the culture medium allowed the counterselection efficacy of the *Ts-sacB* system to be determined. Shifting the temperature from 30°C to 37°C resulted in a two-fold reduction in CFU counts while sucrose alone reduced CFU counts by about two orders of magnitude (Table 3). The combination of both counterselections showed a clear additive effect reducing CFU counts by 3 to 4 orders of magnitude. Thus, although about 10 times less efficient than the counterselection efficiency measured in *M. smegmatis* [16], [42], results were encouraging in that they indicated that, in spite of the relatively narrow temperature growth range of *M. chelonae*, the *Ts-sacB* plasmid could successfully be used to deliver allelic exchange substrates to the cells. This is in contrast to the situation in *M. abscessus* ATCC 19977 where the *Ts-sacB* system was shown not to be functional due to the lack of counterselective efficacy of *sacB* [16]. We thus next proceeded to the transformation of all six (Zeo and Kan) porin knock-out constructs in *M. chelonae* ATCC 35752 and selected for transformants at 30°C on Zeo or Kan plates. One to three XylE^+^ transformants of each were propagated in liquid medium at 30°C in the presence of Zeo or Kan and then serially diluted and plated onto agar plates containing 10% sucrose and Kan or Zeo at 37°C. The outcome of these experiments is summarized in Table 4. Results clearly illustrated the superiority of *zeo* over *kan* in the selection of double crossover mutants. Whereas, 50 to 100% of the sucrose resistant, Zeo^R^ and XylE^−^ clones isolated at the last selection step corresponded to knock-out mutants (Fig. 1B), in none but one case (MCH_4689; transformant T2) did the plating of Kan^R^ transformants yield mutants. The sucrose resistant/Kan^R^/XylE^−^ clones instead most likely corresponded to Kan spontaneous resistant clones that arose during the multiple culturing steps in the presence of Kan. The fact that *MCH_4689* knock-out mutants were isolated upon plating of the pPR27-4689-KX transformant T2 suggests that double crossover events probably occurred earlier in this particular culture relative to the development of spontaneous resistance facilitating the detection of knock-out mutants at the final selection step.

**Table 3:** Counterselection efficiency of the *Ts-sacB* system in *M. chelonae* ATCC 35752.

	Recovered colonies				
Transformant	7H11-OADC	7H11-OADC	7H11-OADC	7H11-OADC	Counterselection
	Zeo (30°C)	Zeo (37°C)	Zeo/Suc (30°C)	Zeo/Suc (37°C)	efficiency (a)
*MCH* (ATCC)	9.7×10^8^	4.1×10^8^	1.6×10^7^	4.9×10^5^	3.38×10^−4^
pPR27-4691-ZX					(+/−1.5×10^−4^)

(a) The experiment was conducted on three independent transformants and mean counterselection efficiencies +/− standard deviations are indicated.

**Table 4:** Comparative efficiency of the *Ts-sacB* system using *zeo* and *kan* disrupted allelic exchange substrates in *M. chelonae* ATCC 35752.

Transformant	% of XylE^−^ Suc^R^	Number of XylE^−^	Number of
	Zeo^R^ or Kan^R^ CFUs	CFUs analyzed by	confirmed double
		PCR	crossover mutants
pPR27-4689-ZX			
T1	100%	4	3
pPR27-4690-ZX			
T1	100%	4	3
T2	11%	4	2
pPR27-4691-ZX			
T1	49%	4	4
T2	29%	4	3
pPR27-4689-KX			
T1	100%	5	0
T2	74%	8	8
T3	100%	5	0
pPR27-4690-KX			
T1	100%	8	0
T2	99%	10	0
T3	100%	8	0
pPR27-4691-KX			
T1	100%	10	0
T2	100%	8	0

One to three transformants (T1, T2 and T3) were selected on plates upon transformation with the pPR27-derived plasmids, grown in 7H9-OADC broth at 30°C for 5 to 7 days, and finally plated onto 7H11-OADC containing Kan or Zeo and 10% sucrose at 37°C. The percentage of CFUs presenting the expected phenotype for allelic exchange mutants at the last selection step of the Ts-SacB procedure (sucrose resistant; Kan^R^ or Zeo^R^ and XylE^−^) is indicated for each construct. Four to ten candidate mutants were analyzed by PCR in each case and the number of double crossover mutants identified is indicated in the last column.

### Construction of allelic exchange mutants using the recombineering system

The same *MCH_4689c*, *MCH_4690c* and *MCH_4691c zeo* cassette-disrupted alleles as the ones used in the *Ts-SacB* system were used in the recombineering system. Care was taken in preparing the linear DNA substrates to generate fragments with incompatible ends that would not recircularize in the bacteria, in order to avoid the selection of single crossover events. All three allelic exchange substrates were electroporated in *M. chelonae* ATCC 35752 harboring the pJV53-xylE plasmid (Table 1) and transformants selected on Zeo plates at 30°C. Twenty-nine (*MCH_4689c*), 65 (*MCH_4690c*) and 57 (*MCH_4691c*) Zeo^R^ colonies were obtained in a typical transformation experiment with 300 ng of linear DNA. The number of transformants, however, increased three-fold when the quantity of linear DNA substrate was increased from 300 ng to 1 µg indicating that saturation conditions had not been reached. Ten to fourteen clones were picked for each gene and propagated in liquid broth prior to genomic DNA isolation. PCR analysis followed by sequencing confirmed that allelic replacement had occurred in 36% of the *MCH_4691c* mutant candidates, 80% of the *MCH_4690c* candidates and 30% of the *MCH_4689c* candidates (Fig. 1B). Therefore, more than 30% of the Zeo^R^ transformants corresponded to allelic exchange mutants, a selection efficiency lower than that reported for *M. smegmatis* (>90%) [16], [20] but greater than that reported for *M. abscessus* ATCC 19977 (7%) [16].

Because of concerns that genetic rearrangements may occur in the knock-out mutants as a result of the retention of the recombinase expression plasmid pJV53-xylE, attempts were then made to cure this plasmid from the porin mutants by culturing on medium devoid of Kan. Interestingly, the direct selection of recombineering mutants on Zeo-containing plates in the absence of Kan yielded Zeo^R^ colonies, 15 to 26% of which were XylE^−^ and Kan-susceptible, indicative of the loss of pJV53-xylE. In the case of knock-out mutants that had retained XylE positivity and Kan resistance, two passages in liquid broth devoid of Kan followed by plating on Zeo-containing agar were sufficient to yield cultures entirely cured of the plasmid. PCR amplification of the *kan* cassette further confirmed that the pJV53-xylE plasmid had been lost from the selected porin mutants. Thus, in the absence of Kan selective pressure, the pJV53-xylE plasmid can efficiently be cured either during the mutant selection step or upon one or two passages of the selected knock-out clones in medium devoid of Kan.

Porin production in each of the mutant strains was analyzed by immunoblotting using polyclonal antibodies raised against the purified MspA protein of *M. smegmatis* [23]. The mature MCH_4691c, MCH_4690c and MCH_4689c porin products have an expected molecular weight of about 19.6 KDa and display about 73% amino acid identity with the mature MspA protein. Of all mutants, the *MCH_4691c* knock-out strain was the one with the lowest porin expression which was also significantly (approximately 50%) less than in the wild-type parent strain (Fig. 1C). In contrast, relative to the wild-type parent, porin production was similar in the *MCH_4689c* mutant (94.4% of the wild-type porin content) and slightly increased in the *MCH_4690c* mutant (130% of the wild-type porin content) (Fig. 1C). Thus, *M. chelonae* appears to compensate for the disruption of *MCH_4690c* and *MCH_4689c* by overexpressing one or two of the remaining porin genes. Complementation of the *MCH_4691c* knock-out mutant with *MCH_4691c* expressed from the replicative multicopy plasmid pOMK restored porin production beyond wild-type levels (Fig. 1C).

### Growth rate, glucose uptake and biocide susceptibility of the *M. chelonae* porin knock-out mutants

**Figure 2:**
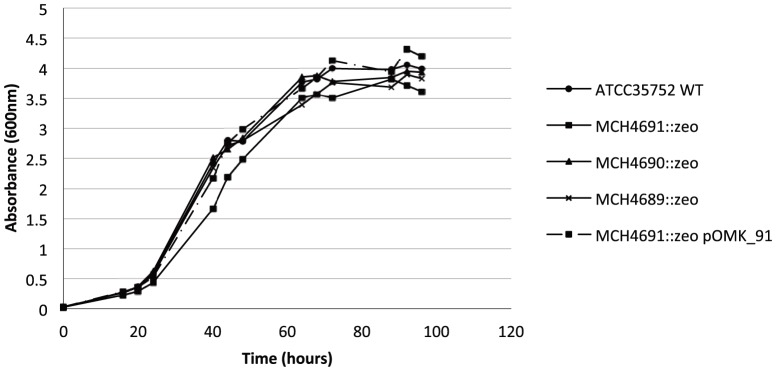
Growth rates of wild-type *M. chelonae* ATCC 35752, its isogenic porin knock-out mutants and complemented *MCH_4691* mutant strains in 7H9-OADC-Tween 80 broth at 30°C. Shown are representative results of two to three independent experiments using different culture batches.

The growth rate of the *MCH_4691c* mutant in 7H9-OADC-Tween 80 broth was slightly but reproducibly decreased compared to the wild-type strain and other knock-out mutants (Fig. 2). Wild-type growth was restored in the *MCH_4691c* mutant upon complementation with *MCH_4691c* expressed from pOMK (Fig. 2).

**Figure 3:**
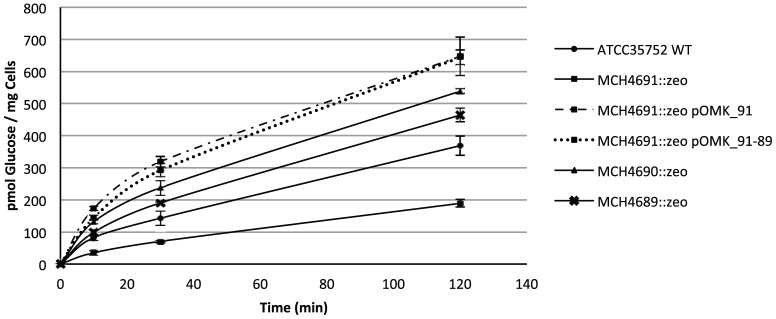
Glucose uptake by *M. chelonae* ATCC 35752 and its isogenic porin knock-out mutants. The accumulation of [U-^14^C]glucose by the strains over time was measured as described under Materials and Methods. Glucose uptake rates were calculated on the first 10 min of the reactions. Uptake experiments were performed in triplicates and are shown with their standard deviations.

Consistent with decreased porin expression, the *MCH_4691c* mutant was also 2.3-fold less proficient at taking up [^14^C]-glucose than wild-type *M. chelonae* ATCC 35752 (Fig. 3). Glucose uptake rates were restored beyond wild-type levels in the *MCH_4691c* mutant complemented with *MCH_4691c* or with the three-porin gene cluster (Fig. 3). The *MCH_4690c* and *MCH_4689c* mutants that produce slightly more or equivalent amounts of porins as the *M. chelonae* ATCC 35752 strain (Fig. 1C) consistently showed 1.6 and 1.2-fold increased [^14^C]-glucose uptake rates compared to their wild-type parent.

The wild-type and mutant strains, however, did not differ in terms of their susceptibility to tetracycline (MIC = 16–32 µg/ml), ethambutol (MIC = 8–16 µg/ml), chloramphenicol (MIC = 8–16 µg/ml), erythromycin (MIC = 4–8 µg/ml), linezolid (MIC = 8–16 µg/ml), and rifampicin (MIC = 256 µg/ml).

Altogether, the results suggest that MCH_4691c is the main porin of *M. chelonae* ATCC 35752 under the culture conditions used in this study. That polar effects of *MCH_4691c* disruption on the expression of downstream porin genes account for this result is unlikely given the 115-bp of intergenic space separating *MCH_4691c* from *MCH_4690c* and the 222-bp separating *MCH_4690c* from *MCH_4689c*. The presence of an A/T-rich region 60 bp upstream from the start codon of *MCH_4690c* further supports the existence of a promoter region upstream this gene.

## Conclusions

In conclusion, both the *Ts-sacB* and the recombineering homologous recombination systems can be used to inactivate genes in *M. chelonae* as long as efficient antibiotic resistance selection markers are used. The genetic methodologies described here open the way to the genetic dissection of key aspects of the physiology, biocide resistance and virulence of *M. chelonae*. The detailed characterization of the roles of each of the three porins of *M. chelonae* ATCC 35752 in the physiology and virulence of this bacterium is out of the scope of the present work but further experiments have begun in our laboratory to study their expression and regulation, and the effects that their combined inactivation might have on bacterial growth, biocide susceptibility and virulence.

## Supporting Information

Figure S1 **Details of the gene replacement protocols used in** ***M. chelonae*** **ATCC 35752.** See text for further details.
(PDF)

Figure S2 **Sequence alignment of the three porins from** ***M. chelonae*** **ATCC 35752.**
(PDF)
