# Supplementary figures and images for: Gene Replacement in Mycobacterium chelonae: Application to the Construction of Porin Knock-Out Mutants
Source: PLoS One. 2014 Apr 16;9(4):e94951. doi: 10.1371/journal.pone.0094951 (PMC3989263; doi:10.1371/journal.pone.0094951)

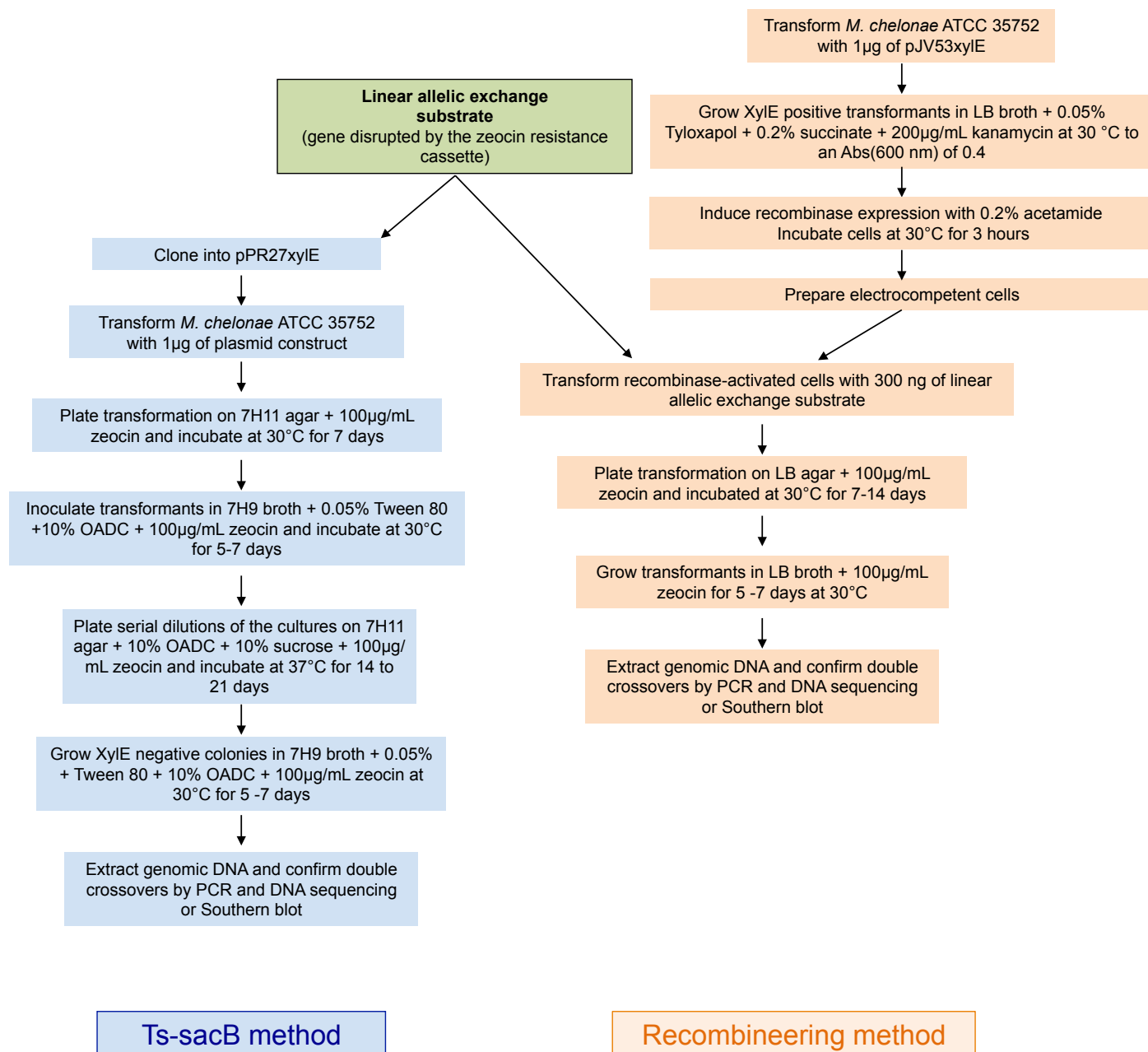

Figure S1

Supplement: Figure S1 — Details of the gene replacement protocols used in M. chelonae ATCC 35752. See text for further details. (PDF) [file pone.0094951.s001.pdf]
