# Supplementary material for: Gene Replacement in Mycobacterium chelonae: Application to the Construction of Porin Knock-Out Mutants
Source: PLoS One. 2014 Apr 16;9(4):e94951. doi: 10.1371/journal.pone.0094951 (PMC3989263; doi:10.1371/journal.pone.0094951)
Supplement: Figure S2 — Sequence alignment of the three porins from M. chelonae ATCC 35752. (PDF) [file pone.0094951.s002.pdf]

<u>Fig. S2</u>: Sequence alignment of the three porins from *M. chelonae* ATCC 35752.

The arrow denotes the position of the cleavage site of the signal peptides as predicted by SignalP 3.0.

```
           1        10        20        30        40        50        60        70        80        90        100       110       120       130
           |--------+---------+---------+---------+---------+---------+---------+---------+---------+---------+---------+---------+---------|
MCH4691c   MKLLSKVSGWARHGLLAVGALVMTLVALFATAATAHAGLDDELTLVDGKGRTLRIQQWDTFLNGVFPLDRNRLTREWFHSGRAVYEVTGPGADAFEGTLELGYQVGYPWSLGVGLNFNYTTPNTSILYGI
MCH4690c         MRGGVRRPLQWLAAVFMLVVMAFTATATAHAGLDDELTLVDGKGRTLRIQQWDTFLNGVFPLDRNRLTREWFHSGRAVYEVTGPGADAFEGTLELGYQVGYPWSLGVGLNFNYTTPNTSILYGI
MCH4689c   MRTGGIWQVMHNTLMRLVLAVAGVVGLAVIGTATAHAGLDDELTLVDGKGRTLRIQQWDTFLNGVFPLDRNRLTREWFHSGRAAYEVTGPAADTFEGTLELGYQVGYPWSLGVGLNFNYTTPNTSILYGI
Consensus  m.......g..r..$..v.Av.m.vv.af..tATAHAGLDDELTLVDGKGRTLRIQQWDTFLNGVFPLDRNRLTREWFHSGRAvYEVTGPgADaFEGTLELGYQVGYPWSLGVGLNFNYTTPNTSILYGI

           131      140       150       160       170       180       190       200       210       220 224
           |--------+---------+---------+---------+---------+---------+---------+---------+---------+---|
MCH4691c   PNAFGGNPEASYIQTTNLLPSAGINVDLGNGPGIQEVATFSVAIAGPKGAVAVSNAHGTVTGAAGGVLLRPYARLISSAGDSVTTYGETWDMKX
MCH4690c   PNAFGGGPEASYIQTTNLLPSAGINVDLGNGPGIQEVATFSVAIAGPKGAVAVSNAHGTVTGAAGGVLLRPYARLISSAGDSVTTYGETWDMKX
MCH4689c   PNAFGGGPEASYIQTTNLLPSAGINVDLGNGPGIQEVATFSVAIAGPKGAVAVSNAHGTVTGAAGGVLLRPYARLISSAGDSVTTYGETWDMKX
Consensus  PNAFGGgPEASYIQTTNLLPSAGINVDLGNGPGIQEVATFSVAIAGPKGAVAVSNAHGTVTGAAGGVLLRPYARLISSAGDSVTTYGETWDMKX
```
